# Are single odorous components of a predator sufficient to elicit defensive behaviors in prey species?

**DOI:** 10.3389/fnins.2015.00263

**Published:** 2015-07-29

**Authors:** Raimund Apfelbach, Michael H. Parsons, Helena A. Soini, Milos V. Novotny

**Affiliations:** ^1^Animal Physiology, Institute for Neurobiology, University of TübingenTübingen, Germany; ^2^Department of Biology, Hofstra UniversityHempstead, NY, USA; ^3^Department of Chemistry, Institute for Pheromone Research, Indiana UniversityBloomington, IN, USA

**Keywords:** predator odors, aging of odors, predator naive prey, odor avoidance, field studies in Australia

## Abstract

When exposed to the odor of a sympatric predator, prey animals typically display escape or defensive responses. These phenomena have been well-documented, especially in rodents, when exposed to the odor of a cat, ferret, or fox. As a result of these experiments new discussions center on the following questions: (1) is a single volatile compound such as a major or a minor mixture constituent in urine or feces, emitted by the predator sufficient to cause defensive reactions in a potential prey species or (2) is a whole array of odors required to elicit a response and (3) will the relative size or escapability of the prey as compared to the predator influence responsiveness. Most predator-prey studies on this topic have been performed in the laboratory or under semi-natural conditions. Field studies could help to find answers to these questions. Australian mammals are completely naïve toward the introduced placental carnivores. That offers ideal opportunities to analyze in the field the responses of potential prey species to unknown predator odors. During the last decades researchers have accumulated an enormous amount of data exploring the effects of eutherian predator odors on native marsupial mammals. In this review, we will give a survey about the development of olfactory research, chemical signals and their influence on the behavior and—in some cases—physiology of prey species. In addition, we report on the effects of predator odor experiments performed under natural conditions in Australia. When studying all these literature we learned that data gained under controlled laboratory conditions elucidate the role of individual odors on brain structures and ultimately on a comparatively narrow range behaviors. In contrast to single odors odor arrays mimic much more the situation prey animals are confronted to in nature. Therefore, a broad range of methodology—from chemistry to ecology including anatomy, physiology, and behavior—is needed to understand all the different (relevant) stimuli that govern and guide the interactions between a predator and its potential prey.

## Development of olfactory research

Chemical ecology is one of the most fascinating themes in modern biology. Chemical compounds are essential in intraspecific communication as well as in information exchange between different species. Chemical signals are involved in the defense of prey species against predators, competitors, parasites, microbes, and other potentially harmful organisms (Derby and Aggio, 2011). In short, the challenge in chemical ecology is to demonstrate how chemically mediated interactions steer ecology and evolutionary processes at all levels of ecological organization (Vet, 1999).

Among the first scientific reports on chemicals secreted by a carnivore was the paper by Albone and Perry (1975). The authors analyzed the anal sac secretion of the red fox (*Vulpes vulpes*) and of the lion (*Panthera leo*). The major components of red fox urine deposited in their tracks on snow during the mating and breeding season were later structurally identified (Jorgenson et al., 1978). It was noted that the characteristic “skunk-like odor” of these fox tracks is likely due to sulfur-containing constituents of urine. The synthetic blends of the major urinary constituents were made and deposited in the fox natural habitat to test their ecological significance (Whitten et al., 1980; Wilson et al., 1980). Seasonal variations in the excretion of captive fox urinary volatiles were further investigated (Bailey et al., 1980). During the 1970s and early 1980s, chemical constituents of the defensive secretions of the striped skunk (*Mephitis mephitis*) were also positively identified as mainly sulfur-containing compounds (Andersen and Bernstein, 1975; Andersen et al., 1982).

During roughly the same time period, the chemist Schildknecht and his coworkers analyzed the anal gland secretions of several mustelid species including the mink (*Mustela vison*), the polecat (*Mustela putorius*) and the badger (*Meles meles*) (Schildknecht et al., 1976, 1981; Schildknecht and Birkner, 1983; Schildknecht and Hiller, 1984). Considering the limited analytical capabilities at that time, these studies are still among the most detailed reports on semiochemicals in carnivores. The authors reported 3,3-dimethyl-1,2-dithiolane, 2,2-dimethyl-, cis- and trans-2,3-dimethyl-, 2-propyl-, and 2-pentylthietane characteristic to the polecat (*Mustela putorius* L.) and the ferret (*Mustela putorius furo*). In the odorous secretion of the stoat (*Mustela ermine*) they found 2-methyl-, 2-opyl-, and 2-pentylthietan. Similar sulfur-containing components were found in the secretion of the anal glands of weasel *(Mustela nivalis*). On the contrary, no sulfur-containing compounds were observed in the beech-marten (*Martes foina*) and the pine-marten (*Martes martes*) odoriferous secretions (Schildknecht and Birkner, 1983).

As mustelids became more popular in scientific research, more research groups focused on the anal secretions of mustelid species (Crump, 1980; Brinck et al., 1983; Crump and Moors, 1985). The general ideas about the importance of chemical communication in mammals were significantly advanced by the first reports of chemical structures of primer pheromones in rodents (Jemiolo et al., 1986; Novotny et al., 1986, 1990) starting in the 1980s. Importantly, the analytical methodologies developed for this research became a major stimulus for broad studies of ecological significance. All these studies have opened avenues for biologists to investigate chemical communication within individual species and the relationship between predators and prey.

The chemical compounds emitted by a species and the effects they might cause in another species became hot spots of scientific interest. Among the first to launch this research line were Nolte et al. (1994). They sparked a field of highest scientific interest when they published their paper “why are predator odors aversive to prey?” This question is still investigated and debated by biologists, chemists, and ecologists. Until now, no one has introduced an adaptive framework to organize the data and speculations for the broad range of varying results. We will propose a novel adaptive framework to compare the overall risk that a prey animal might accept against the degree of danger represented by one or more predator cues. The most obvious indicator of risk relates to how likely a single encounter of predator-prey would result in foregone feeding or mating opportunities, serious injury, or demise e.g., the ability of the prey to defend, suppress, or survive the encounter. For this reason we propose a likelihood of risk category by the relative size or elusiveness of the prey as compared to the predator (supplement, Table 1).

**Table 1:** **Literature review of chemical based predator–prey studies from Australia with foci on the source cue, integrity of signal and outcome measured**.

**Time Period**	**Literature referenced**	**Predator species**	**Prey species**	**Variations of chemical**	**Response measured**	**Risk category**
2000–2005	Head et al., 2002	White-lipped snake odors (*Drysdalia coronoides*) from damp paper towel in snake enclosure	Mountain log skink (*Pseudemoia entrecasteauxii*) paper-towel and rocks from cage	Not stated	Shift in habitat use	4
	Blumstein et al., 2002	Feces from red fox (*Vulpes vulpes*), kodiak bear (*Ursus arctos*) and dingo (*Canis L. dingo*) placed beneath feeding tray	Tammar wallaby (*Macropus eugenii*) and rednecked pademelon (*Thylogale thetis*)	Frozen at −20°C	No changes in foraging behavior	2
	Downes, 2002	Yellow-faced whip snake (*Demensaina psammophis*) paper towel and rocks from cage	Common garden skink (*Lampropholis delicata*)	Not stated	20% reduction in mobility of prey	4
	Banks et al., 2003	Domestic dog feces (*Canis domesticus*)	Bush rat (*Rattus fuscipes*)	Fresh	No influence on trapping success	2
	Powell and Banks, 2004	Fox feces (*Vulpes vulpes*)	House mouse (*Mus musculus*)	Fresh	No change in food removed (GUD^*^)	4
	Ramp et al., 2005	Synthetic dog (*Canis domesticus*) urine	Parma wallaby *(Macropus parma*) and red-necked pademelon (*Thylogale thetis*)	Not stated (synthetic used)	*T. thetis* investigated scent more, *M. Parma* showed aversive response	4
	Russell and Banks, 2005	Red fox (*Vulpes vulpes*), tiger quoll (*Dasyurus maculatus*)	Northern brown bandicoot (*Isoodon macrourus*) and brushtail possum (*Trichosurus vulpecula*)	Stated fresh or frozen	Captured significantly more often in traps scented with tiger quoll odor	4, 3
2006–2010	Hayes et al., 2006	Varied: carpet python, dingo, quoll, red fox (*Vulpes vulpes*)	Fawn-footed melomys (*Melomys cervinipes*), bush rat, (*Rattus fuscipes*), giant white-tailed rat (*Uromys caudimaculatus*)	Frozen at −20°C in airtight vials with Teflon-lined lids	During dry season all species avoided all predator odor stations	4
	Murray et al., 2006	Tiger feces *(Panthera tigris*)	Goat (*Capra hircus*)	Feces mixed in bentonite (>dispersal)	Reduced feeding	2
	Parsons et al., 2007	Coyote (*Canis latrans*), dingo (*Canis L. dingo*)	Western gray kangaroo (*Macropus fuligonosus*)	Pooled urine (12–16 adult males) replenished each trial	Increased GUD and flight/startles for dingo as compared to coyote	2, 3
	Russell and Banks, 2007	Tiger quoll (*Dasyurus maculatus*) and the introduced placental red fox (*Vulpes vulpes*)	Bush rat (*Rattus fuscipes*), swamp rat (*Rattus lutreolus*), eastern chestnut mouse (*Pseudomys gracilicaudatus*), brown antechinus (*Antechinus stuartii*)	Stated fresh and/or frozen	Native rodents more likely trapped in control than treatment. *Antechinus* showed no trapping differential	4, 2
	Lloyd et al., 2009	Goanna (*Varanus tristis*) a species that consumes skinks as a major proportion of its diet, and *V. varius*, which does not	Tropical skink (*Carlia rostralis*), (*C. rubrigularus*) and (*C. storri*)	Filter paper dampened water	Two of the three species avoided *V. tristis*. None avoided *V. varius*	4, 2
	Cox et al., 2010	Feces from tiger (*Panthera tigris*) and tasmanian devil (*Sarcophilus harrisii*)	Goat (*Capra hircus*) and eastern gray kangaroo (*Macropus giganteus*)	Feces mixed in a bentonite carrier (aids in dispersal)	Both prey species avoided odors from predators that had fed on these species prior to trials (diet specific response)	2, 2
	Parsons and Blumstein, 2010a	Dingo (*Canis l. dingo*) urine; feces	Western gray kangaroo(*Macropus fuliginosus*)	Pooled urine/feces (12–16 adult males) repolished trial	Flight; = GUD	3
	Parsons and Blumstein, 2010b	Dingo urine (*Canis l. dingo*)	Wallabies (Macropus rufogrisius, pademelon (*Thylogale billardierii*), brush-tailed possum (*Trichosurus vulpecula*)	Maintained fresh	Flight; = GUD	2,2
2011–2015	Nersesian et al., 2012	Fox (*Vulpes vulpes*), owl	Brush-tailed possums (*Trichosurus vulpecula*)		< feeding time, < vigilance varied with indirect cues	4
	Anson and Dickman, 2013	Red fox (*Vulpes vulpes*) in areas where fox impacts had been greatest, and to cues of the native lace monitor (*Varanus varius*)	Common ringtail possum (*Pseudocheirus peregrinu*	Collected fresh, 1 part feces mixed with 5 parts water	Flight alarm calling to both odors	4, 3
	Bytheway et al., 2013	Dog/dingo hybrid integument odor collected on towels	Black rat (*Rattus rattus*)	Fresh	Increased GUD, visitation/investigation	4
	Descovich et al., 2012	Dingo feces	Southern hairy nosed wombat (*Lasiorhinus latifrons*)	Frozen at −20°C	When feces were present, the wombats used concealed locations more often than other periods	4
	Cremona et al., 2014	Dingo (*Canis l. dingo*) and the northern quoll (*Dasyurus hallucatus*) feces	Rock rat (*Zyzomys* spp.)	Not stated	Rock rats demonstrated a stronger avoidance to quoll odor than to dingo odor	3
	Mella et al., 2014a	Fox (*Vulpes vulpes*) and dingo (*Canis L. dingo*) feces	Western gray kangaroo (*Macropus fuliginosus*)	Fresh	Modifying space use by rapidly escaping from both odors	3, 3
	Mella et al., 2014b	Domestic dog (*Canis domesticus*) urine, owl pellets	Brushtail possum (*Trichosurus vulpecula*)	Urine used within 24 h after collection	Possums reacted more strongly to indirect cues (no change to direct)	3, 3
	Spencer et al., 2014	Fox (*Vulpes vulpes*) and cat (*Felis catus*) urine	*Notomys alexis*, a terrestrial native rodent	Dowels were soaked overnight in predator urine stored at 1°C	No effect, prey relies on escape	4

*Outcomes based on presumed risk category of species. Risk category: 1, lowest risk; 2, significant size differential, but uncommon predation (non-historic); 3, lethal predator, but comparatively large prey; 4, highest risk of lethality with direct predation common*.

* *GUD = the weight of food that animals leave behind next to treatments, that they otherwise would have consumed in the control tray. Search string = “predator prey scent odor Australia”; search range 2000–2005, 2006–2010, 2011–2015. Inclusive of all experimental journal articles with terrestrial vertebrate field studies involving predator wastes as a source cue including: urine, feces, dander, or integumentary*.

Implicit in our model is an understanding that smaller, more vulnerable, prey may respond aversively to single-molecule odors while larger, or most elusive, prey may attenuate their response according to composite molecules that convey additional information about the degree of risk inherent in the scent. For instance small kangaroos and wallabies may respond aversively to domestic dog urine, predators they have had no evolutionary contact with—or possibly even aged, chemically degraded scents. However, larger macropods such as red kangaroos, may not respond to a single compound. They likely require complex scents that include additional information on the size and recentness of void from the predator, before foregoing mating opportunities or leaving a food patch. It would after all, be evolutionarily disadvantageous for a 50+ kg herbivore to respond to wastes secreted by a 700 g, predator.

Much of the present research concentrates on three basic topics: (1) How effective are carnivorous chemical compounds as repellents to prey species of different size, (2) do such chemical compounds suppress breeding success in prey species and (3) which brain structures of the prey species are involved in the avoidance/fear responses. A comprehensive review of field and laboratory studies about the positive and negative effects of predator odors on mammalian prey species has been reported in detail (Apfelbach et al., 2005). The involvement of brain structures in the avoidance/fear responses has been recently very well-addressed by Takahashi (2014).

The effects of predator odors or individual compounds in a complex odor source can be evaluated in the laboratory and in the field. Besides natural predator odors, synthetic predator semiochemicals (Lindgren et al., 1995) and—in another study—a range of seven predator odors and, in addition diesel oil, as repellents for wildlife have been used in field studies (Engelhart and Müller-Schwarze, 1995). In that study, coyote (*Canis latrans*), lynx (*Lynx canadensis*) and river otter (*Lutra canadensis*) odors had the strongest effects, while diesel oil was effective too, but the effects were weaker. So far, no field data are available about the effectiveness of a predator odor to suppress or at least to reduce the reproduction success of potential prey species.

In our review the seminal discussion centers mainly on two questions: (1) is just one single volatile compound in, for example, urine or feces emitted by the predator enough to release defensive reactions in a potential prey species—or must there be a whole profile of odors? (2) Do prey animals respond innately to olfactory predator cues? Our focus is set mainly on mammals, but other vertebrates and lower taxa will be included on some occasions.

## Composition and aging of olfactory signals

An odorant is a chemical compound that gives a particular smell to a source. An odor is typically viewed as a volatile molecule, or a set of molecules, that convey some information about the sender to a receiver; generally, the molecules meeting this specification have molecular weights lower than 300 Da.

In mammals, there are different bodily odor sources. Besides urinary odors, other odors emitted from other sources by an animal may serve in the behavioral context to convey information such as degree of hunger or satiety, single, or multiple predators (from over-marking) and importantly, the size, and specificity of the predator- especially important to prey that are otherwise large enough or elusive enough to live comfortably among predators that convey little risk. Among those are anal gland secretions, fecal odors, and vaginal secretions, just to mention the most obvious ones. Fur, dander, sebum, saliva, and tears can also transmit “infochemicals.” Some species possess additional glands like the preorbital glands and tarsal organs in many hoofed animals or the supplementary sacculi (located at the opening of the cheek pouches) and the midventral gland of dwarf hamsters (*Phodopus* spec.) secreting substances of relevance for intraspecific communication.

In invertebrates, just one molecular entity is often sufficient to transmit an important message from one individual to another; the best known example is the pheromone bombykol produced by the silkworm *Bombyx mori* (Schneider et al., 1968; Kaissling, 2014). Among insects, pheromones (intraspecific messengers) are typically composed of one or only very few molecular types such as hydrocarbons in insect cuticles (Blomquist and Bagnères, 2010), while most odorous substances secreted by mammals are typically composed of numerous different volatile compounds. Depending on the species, up to 70 or more volatile compounds have been found. For example, in ferret (*Mustela putorius furo*) urine, 31 volatile urinary compounds have been identified and compared with 26 anal gland compounds of the same species. Only 10 compounds were found common to both sources (Zhang et al., 2005). The two marking sources likely convey different messages to conspecifics. This possibility is backed by our observations on wild living ferrets and polecats (*Mustela putorius*). Males and females urinate all over their territories, but defecate only on specific spots. While urine and fecal odors have putative functions in intraspecific communication, the secretions of the anal glands (containing high amounts of sulfur compounds) seem to serve a dual purpose for defense: when cornered or threatened, and as “alarm pheromones” to warn conspecifics of imminent danger. Two simple compounds from the peri-anal gland, 4-methylpentanal and hexenal, function together as the alarm pheromone in Norway rats (*Rattus norvegicus*) (Inagaki et al., 2014). These two molecules lose their function, when acting alone.

The composition of the urine odors and possibly also of the anal gland odors may change during the seasons in accordance with the endocrine status and also the diet consumed by a predator. This was seen in the elevated seasonal levels of isopentyl methyl sulfide in the red fox urine (Bailey et al., 1980). Several volatile compounds showed peak levels also in the wolf urine (*Canis lupus*) depending on the season. These included isopentyl methyl sulfide and several carbonyl compounds (Raymer et al., 1984). Hormone treatment experiments later confirmed that testosterone increased wolf urinary volatile compound levels (Raymer et al., 1986).

Volatile compounds in urine or in a secretion differ in molecular weights and physicochemical properties, such as boiling points, vapor pressure, and solubility properties in water. For instance, in the ferret urine (Zhang et al., 2005) the molecular weight of the individual molecules ranged from 60.05 Da (acetic acid; boiling point: 118°C; vapor pressure: 1290 Pa) to 256.42 Da (hexadecanoic acid; boiling point: 215°C; vapor pressure: 0.5 × 10^−4^ Pa). In the urine of another carnivore (Osada et al., 2013), the wolf (*Canis lupus*), the molecular weight range extends from 62.13 Da (dimethyl sulfide; boiling point: 35°C; vapor pressure 53,700 Pa) to 122.12 Da (benzoic acid; boiling point: 249°C; vapor pressure 0.1 Pa). In general, compounds with low molecular weights, low polarity, and high vapor pressure evaporate faster than the “heavier,” more polar compounds with low vapor pressure properties. These differences might explain why over time the composition of a secretion changes. We will call it “aging of a signal.” In this context, aging can mean decreased concentrations or a loss of some components and/or changing ratios between the compounds. This process would be particularly prominent in the arid conditions of Australia, where all compounds, regardless of molecular weight, evaporate more quickly due to increasing of vapor pressures at elevated environmental temperatures. Another consideration is the water solubility of the compound in environmental conditions. Compounds with low water solubility will be less affected by rain and therefore might resist environmental stress much longer than compounds with high water solubility. The much less volatile components of urinary marks and secretions, such as lipids and proteins, may also retain and slowly release volatile chemosignals into the environment.

The numbers of research papers on aging of urine or gland secretion odor compounds are very limited. In contrast to studies on chemical signals in animals, aging has been intensively investigated in food chemistry and interesting results have been reported. For instance, the flavor characteristics of beer appear to deteriorate greatly with time (Gijs et al., 2002). Similarly, changes in some volatile constituents of brandy (Onishi et al., 1977) and sherry wines have been reported (Munoz et al., 2007). Among the first to report on behavioral effects of aged urine were Coppola and Vandenbergh (1985). These authors determined how long the puberty delay causing chemosignal emitted from an adult female mouse urine remains active to suppress puberty in young females. According to their data, within 7–10 days after collecting urine, the sample will lose its pheromonal potency to delay puberty in recipient females. The authors suspected that the puberty delay causing signal would lose its potency more rapidly in nature than under laboratory conditions due to the destructive influences of the natural elements.

Neuroendocrine and behavioral responses of mice to urine samples from conspecific males and females, which had aged for different time periods, revealed that the quality and intensity of signaling molecules in urine changed over time (Kwak et al., 2013a). In another study, volatile organic compounds in fresh and aged human urine samples were analyzed and compared (Kwak et al., 2013b).

**Figure 1:**
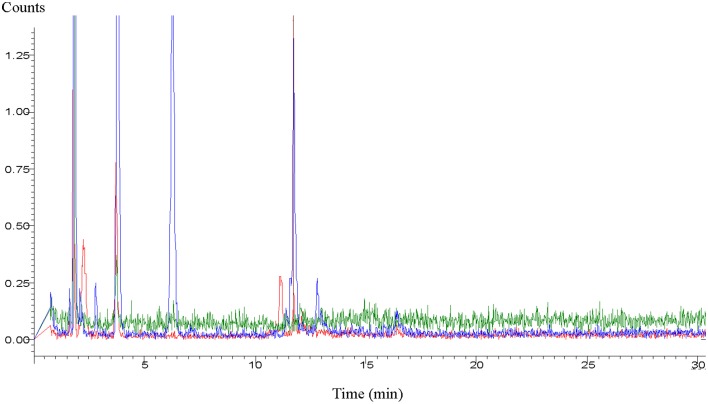
**Aged profile of dingo (***Canis lupus dingo***) urine**. Blue loop refers to fresh male urine, red loop refers to fresh female urine, and green loop refers to male and female urine aged >3 months (Graph supplied by M.H. Parsons).

Due to such unpredictable environmentally-induced changes, researchers run experiments with either fresh secretions or with secretions which were deep-frozen until use. Yet, in spite of deep freezing, the secretions can still undergo composition or conformational changes. This has been convincingly demonstrated in a very recent study during which cat feces was stored at −70°C (Hegab et al., 2014). The statement of these researches reports best the effects of aged chemicals: “Behavioral and hormonal responses and changes in the level of medial hypothalamic c-fos nRNA were examined in Brandt's voles (*Lasiopodomys brandtii*) exposed to the feces of a domestic cat (*Felis catus*) stored for different periods. One hundred voles were tested in the defensive withdrawal apparatus. The voles showed an aversion to freshly collected cat feces, indicated by high levels of flight-related behaviors, increased freezing behavior, and more vigilant rearing compared to old feces. The serum levels of adrenocorticotropic hormone and corticosterone significantly increased when the voles were exposed to fresh cat feces. The level of c-fos mRNA in the medial hypothalamus region was highest in the individual exposed to fresh cat feces. All these behavioral, endocrine, and c-fos-mRNA responses were lower when voles were subject to older cat feces” (Hegab et al., 2014). In a study working with meerkats (*Suricata suricatta*), similar results were found. The freshness of the presented wolf (*Canis lupus*) urine increased vigilance in the prey animals while the increased quantity of urine sample did not cause the similar effect (Zöttl et al., 2013). Similarly, in comparisons of the effects of fresh and previously frozen female mouse urine on male mice, fresh urine triggered stronger courtship ultrasonic vocalization in males (Hoffmann et al., 2009). Gas chromatographic analyses of dingo urine convincingly demonstrated changes in the male and female urine after an aging period of about 3 months or even less (Figure 1).

Data from such studies indicate that very careful experimentation and interpretation of the data is needed when stored biological compounds are used to study behavioral and physiological parameters. It cannot be excluded that in natural environments, loss of response to a predator odor over time is not caused by habituation, but rather aging of the signal.

## Single odors or odor arrays?

Sensing the chemical warnings present in the environment is essential for species survival. Brunswik (1956) suggested the concept of a transient fear scent operating in mice. Emitted by both males and females, this fear scent can be elicited by a single stressful event. In the natural setting, a mouse's tendency to withdraw from sites possessing the fear scent of conspecifics may well-protect mice from predators and other dangerous situations. Similarly, Carr et al. (1970, 1971) reported that male mice avoided an olfactory signal emitted by stressed male and female mice. It was also demonstrated that male mice were repelled by the odor from shocked males and attracted to the odor from non-shocked males (Colyer (1971). Although additional odor sources may be involved, it is believed that a fear scent is contained in the urine (Müller-Velten, 1966). These findings marked the beginning of considering urine as a transmitter of danger odor signals.

Nolte et al. (1994) suspected that predator odors are aversive to prey species due to the high concentrations of sulfurous components (metabolites of protein digestion) in their urine. As reported above, urine or fecal odors are composed of numerous different compounds. Subsequently, many studies in behavioral research have used such complex mixtures and reported the results under these conditions. It is not in the interest of a predator to deposit a signal that persists over longer time periods to warn potential prey about predator's presence. While there is a trade-off involved when predators deploy odors in the environment, it is beneficial for conspecifics to be able to detect these “chemical bulletin boards” for as long as possible. However, the longer the scent is viable, the more likely potential prey could intercept the signal and will respond innately to these kairomones (chemical messages of another species) or will learn to recognize and respond accordingly to these signals. Cheetahs may have evolved the ability to secrete an odorless (elemental) sulfur complex (Burger et al., 2006). On the other hand, secretions may inform conspecifics about a territory owner or a sexual partner ready to mate. In this case, it would be advantageous for the sender when the chemical signal remains in place for a longer time period.

Most mammals were long assumed to have two olfactory systems working independently, the primary olfactory epithelial tissue (MOE) and secondary vomeronasal mediated system (VMO; e.g., pheromones), with the molecular weight of volatile constituents determining the appropriate binding site. This model seemed to have been validated in mice (Trinh and Storm, 2003), but has been since updated to consider the shared role of both organs, now seen to complement one another due to common V1R receptors, and shared processing regions in the amygdala (Kendrick, 2014).

It remains unclear whether single molecule constituents are more or less likely to be received by the primary or secondary systems, than composite chemicals. However, we may operate under the reasonable assumption that predator-secreted compounds are more likely to be detected by the VMO, than foul smelling, or pungent, artificial odors such as diesel fuel, ammonia or feces from non-predatory omnivores (e.g., pigs). These foul odors would contribute less biologically meaningful information and would thus be more likely to be interpreted by the MOE.

In vertebrates, at least in mammals, generally no single compound is known to convey complex behavioral messages between individuals. Yet, examples that a “bouquet” of odors makes up the message, have been reported with red fox (Whitten et al., 1980) and mice (Jemiolo et al., 1986; Novotny et al., 1986; Ma et al., 1998). However, several papers state that some compounds in urine or fecal secretions are especially effective and will alone, or in combination with other molecules, elicit defensive reactions in prey species and/or suppress breeding success. Some of the four most discussed molecular types include:

1. **Pyrazines** are known as volatile nitrogen-containing odoriferous compounds present in vertebrates, plants, insects, fungi, and bacteria (Woolfson and Rothschild, 1990). Pyrazines, such as 2,5-dimethylpyrazine (2,5-DMP), seem to have redundant message contents in vertebrates. In the female mouse urine, 2,5-DMP is involved in a puberty inhibition signaling from a female mouse to other female mice together with five other adrenal-mediated urinary metabolites (Andreolini et al., 1987; Ma et al., 1998).
  Male wolf (*Canis lupus*) urine samples contained more than 50 compounds in the GC-MS analyses with some significant differences in compound levels (Osada et al., 2013). Such differences may allow, for instance, individual recognition within a wolf pack. However, the urine of all three wolfs contained pyrazine derivatives as the predominant active components capable to induce avoidance and freezing behaviors in mice. Osada further reported that the combination of 2,5-DMP and two other pyrazines, which are present in the wolf urine, induced freezing behavior in mice, which was a similar response as to wolf urine, while each pyrazine compound alone was inactive. In the ferret urine, pyrazines could play a role in the odor-sensing and caution expressed by hamsters to the ferret urine (Apfelbach et al., 2015).
2. **2-Phenylethylamine (PEA)** (molecular weight, Mw: 121.18) is a component common to many carnivore odors. It also has been found in non-carnivorous species at much lower concentrations (Ferrero et al., 2011). Like pyrazines it is a moderately volatile compound (vapor pressure 35 Pa, Mokbel et al., 2009), therefore, probably not very effective over longer periods of time. In behavioral studies, rodents avoided a PEA odor source similarly as they avoided predator urines. To verify that PEA is the decisive chemical for the avoiding reaction, the researchers experimentally depleted lion urine of this compound. In the subsequent behavioral experiments, rats showed significant avoidance behavior to 10% content in lion urine, but not to 10% PEA-depleted urine specimen. Aversion was fully restored to a 10% PEA-respiked lion urine. The authors interpret from these data that PEA is a key component of a carnivore odor blend detected and avoided by rodents (Ferrero et al., 2011). However, studies under natural conditions have not been reported.
3. **2,3,5-Trimethyl-3-thiazoline (TMT)** (Mw: 129.22) is found in fox feces (Vernet-Maury, 1980). Over the years, several laboratories reported fear-like alterations in rat behavior due to exposure to synthesized TMT (e.g., Wallace and Rosen, 2000). Physiological data as well as data on brain structures involved in the TMT-elicited defensive responses of rats have been also described (Fendt et al., 2003, 2005; Endres et al., 2005; Dielenberg et al., 2001). The view of TMT as a biologically relevant olfactory stimulus has been challenged. According to Morrow (Morrow et al., 2002), a fear-like biochemical and behavioral response in rats to TMT odor depends on the exposure environment. Others have even failed to observe fearful behaviors (McGregor et al., 2002); since TMT has an acrid, irritating, and powerfully repugnant odor (at least to humans), its effects are more characteristic of an aversive odor, presumably working through a nociceptive mechanism. According to Staples and McGregor (2006), differences in response to TMT and cat odor could depend on the rat strain.
  Blanchard regarded TMT as follows: “These findings suggest that flight/avoidance, although it obviously may occur as one component of a full pattern of defensive and emotional behaviors, is also somewhat separable from the others. When—as appears to be the case with TMT—it is the major, perhaps only consistent defensive behavior elicited, this may reflect a stimulus that is aversive or noxious but with little ability to predict the presence of threat or danger” (Blanchard et al., 2003). To address this criticism, rats were exposed to TMT following either olfactory bulb removal or trigeminal nerve transection (Ayers et al., 2013). The findings indicate that freezing behavior to TMT requires an intact olfactory system, as indicated by the loss of freezing following olfactory bulb removal. Rats with trigeminal nerve transection freeze normally to TMT, suggesting the olfactory system mediates this behavior to TMT. TMT is an ecologically relevant predator odor useful in experiments of unconditioned fear that is mediated via olfaction and not nociception (Ayers et al., 2013).
  Very few behavioral field experiments using TMT as a repellent have been reported. To reduce feeding damage by voles (*Microtus spec*.) on apple trees in orchards Sullivan et al. (1988a) applied TMT to traps and quantified the number of voles caught in TMT-free traps and in traps scented with TMT. The animals significantly avoided the TMT scented traps. In a similar field experiment using Northern pocket gophers (*Thomomys talpoides*) animals also avoided the TMT odor (Sullivan et al., 1988b). However, when testing the two synthetic predator odors TMT and DMDIT (3,3-dimethyl-1,2-dithiolane) for their possible repelling effects on roof rats (*Rattus rattus*) in Hawaiian macademia nut orchards no clear results became visible. The authors (Burwash et al., 1998) stated “overall we could not detect significant differences or consistent trends in response of rats to DMDIT or TMT in these field trials.”
  Without doubt TMT is effective in eliciting fear and escape responses in rats, although they were naive to foxes and fox feces. Interestingly, TMT has not been found in dog feces (Arnould et al., 1998) and also not in anal gland secretions of dog and coyote (Preti et al., 1976), although both carnivore species are closely related to the red fox. These and other results indicate that TMT may be characteristic for the red fox, but possibly not for other predators.
4. A cat-specific substance is **2-amino-7-hydroxy-5,5-dimethyl-4-thiaheptanoic acid (L-felinine**) (MW: 207.29). Felinine, a putative pheromone precursor, has been identified in the urine of several members of the felidae family including the domestic cat (*Felis catus*) (Hendriks et al., 1995b). Cat urine contains 3-mercapto-3-methylbutan-1-ol, a degradation product of felinine, and a putative cat pheromone. This compound gives cat urine its typical odor and may have a function in territorial marking (Hendriks et al., 1995a; Miyazaki et al., 2006). However, no experimental proof has been provided up to date for this assumption. Felinine is a non-volatile amino acid that requires a close contact (“close contact signal”) for the olfactory perception. To investigate the influence of cat odor on reproductive behavior and physiology in the house mouse, cat urine or 0.05% felinine was directly applied to the bedding of pregnant mice every other day. After having given birth, the total number of offspring was counted as well as the number of pups per female. Exposure of mated females to felinine provoked a pregnancy block in 67.85% female mice, while in the control group a birth reduction of only 17.86% was observed (Voznessenskaya, 2014). Felinine has not been tested outside the laboratory conditions so far.

Rats and mice are averse to the odor of a cat's urine, but after they are infected with the parasite *Toxoplasma gondii*, they are attracted to cat urine. This increases the likelihood of being preyed upon and consequently, infecting the cat (Berdoy et al., 2000). Earlier, a similar decrease in predator avoidance in parasitized mice was reported. Mice infected with the naturally occurring *Eimeria vermiformis* spent a significantly longer time period in the proximity of cat odor, while uninfected mice continued to avoid cat urine. This result indicates that infection with *E. vermiformis* in mice reduces the avoidance of a predator odor through neurochemical systems associated with anxiety involving GABA receptor mechanisms (Kavaliers and Colwell, 1995).

Besides felinine odor, cats emit other chemical signals. Cats mark their territories by rubbing their neck at corners or objects leaving a scent behind (“cat neck odor”). When a rat is exposed to such a scent, it exhibits a strong aversive/flight reaction. When Wistar rats were exposed to a cotton pad wiped on a cat body rubbing location, they showed increased hiding behavior, decreased exploration behavior and reduced stimulus approach and investigation. These defensive responses persisted for up to 4 days following a single stimulus exposure (May et al., 2012). C-fos studies revealed a high activation of the brain structures involved in these fear reactions (Dielenberg et al., 2001). So far no information is published about the compound responsible for the reported behavioral effects.

**Figure 2:**
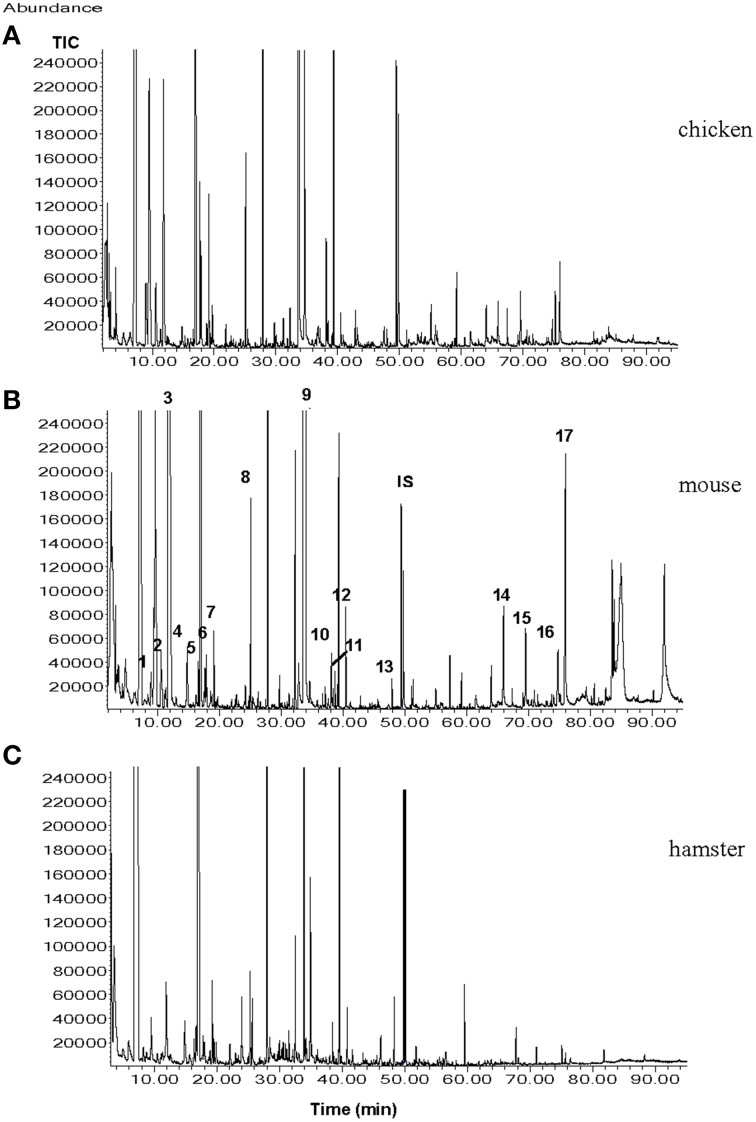
**Total ion chromatograms (TICs) from the male ferret urine samples when ferrets were fed with (A), chicken; (B), mouse; (C), hamster**. Numbers indicate the following compounds 1, xylene; 2, heptanal; 3, 2,5-dimethylpyrazine; 4, benzaldehyde; 5, 6-methyl-5-hepten-2-one; 6, 2,3,5-trimethylpyrazine; 7, 2-ethenyl-6-methylpyrazine; 8, non-anal; 9, quinoline; 10, o-aminoacetophenone; 11, 2-methylquinoline; 12, 2-methylquinazoline; 13, geranylacetone; 14, tetradecanoic acid; 15, pentadecanoic acid; 16, 9-hexadecanoic acid; 17, hexadecanoic acid; IS internal standard (7-tridecanone) (Graph taken from Apfelbach et al., 2015).

It may seem unlikely that only one type of a volatile molecule out of the many compounds (e.g., in urine) is sufficient for eliciting escape or defense reactions in a prey. Considering the above findings, one can conclude that the amount of information encoded in one urinary volatile molecule seems limited. The predator odor information system may act more like a “yes—no” (danger—no danger) information system. Much more information can be encoded in a “bouquet” of several volatile compounds. Already in 1994, Nolte and coworkers reported that herbivorous rodents were able to distinguish between urine collected from coyotes fed cantaloupes vs. those fed a meat diet. Similarly, in a paper by Berton et al. (1998), the authors demonstrated that mice were able to distinguish between the fecal odor of cats subjected to either a vegetarian diet or a carnivorous diet. In a recent study, it was demonstrated that the dwarf hamster (*Phodopus campbelli*) was able to readily distinguish between urine of ferrets fed with chicken, rat, or hamster. While dietary variations are unlikely to result in measurable quantities of additional urinary compounds, the signal-receiving animals may be capable of distinguishing quantitative differences in the urinary volatile compound arrays (shown in Figure 2), and subsequently, perceiving different olfactory messages (Apfelbach et al., 2015).

An interesting question is how fast naïve animals are able to learn to respond to odors of a new, previously unknown predator. Anson and Dickman (2013) explored in a field study the ability of the common ringtail possum (*Pseudocheirus peregrinus*), a semi-arboreal Australian marsupial, to recognize and respond to olfactory cues from the introduced fox (*Vulpes vulpes*). Their results show that in the areas with high fox densities, the selection pressure from the fox has been sufficient for ringtails to develop anti-predator behaviors over a few generations since foxes had become established in the area. In contrast to this finding, no such anti-predator behavior patterns were obvious in the areas where foxes either had not been observed or observed only very recently. A recent paper by Dias and Ressler (2014) will likely receive considerable attention among scientists. These authors examined the inheritance of a parental traumatic olfactory exposure. F0 mice were subject to odor fear conditioning before conception, and subsequently in F1 and F2 generations, an increased behavioral sensitivity to the F0-conditioned odor, acetophenone, but not to other odors, was found. Besides behavioral studies, neuroanatomical, and genetic studies including cross-fostering were employed. Taking all their data into account, the authors concluded that their findings provide a framework for addressing how environmental information may be inherited transgenerationally at behavioral, neuroanatomical, and epigenetic levels.

## Field studies in Australia—a unique experimental field

Australia offers a unique opportunity to observe the relationship between predator-naïve native marsupial prey species of various size and introduced placental predators. This situation allows experiments under natural conditions to follow the question whether predator odors will be ignored or investigated or do they elicit aversive reactions. Researchers have accumulated an enormous amount of data exploring the effects of eutherian predator odors on native marsupial mammals. Some of these data will be included in our review.

The introduction of alien predators often has catastrophic effects on populations of native prey species. Australia offers a unique opportunity to observe the relationship between predator-naïve native prey species of various size and introduced predators. It is a challenge to investigate the “evolution” of a balanced relationship between newcomers and endemic species when there is no consensus on how long predator and prey must remain together in order to co-adapt.

For thousands of years, the dingo was the only eutherian predator well-embedded in the Australian food chain. With the arrival of the Europeans in Australia, previously unknown predator species also arrived at this isolated continent. Predators such as the European fox (*Vulpes vulpes*), the feral cat (*Felis catus*), wild dogs (*Canis lupus familiaris*), coyotes (*Canis latrans*), and ferrets (*Mustela putorius furo*) established themselves and started to threaten naïve native species. Correspondingly, quite a high number of field studies have been reported from Australia. In all these studies, complex natural predator odors and no single volatile compounds were employed.

With a few examples, the relationship between predator and prey will be depicted in this section of the review. Special attention is given to the question as to how effective are unknown carnivorous chemicals as repellents to small (< 2 Kg), medium sized (>2 Kg) and large prey (>10 Kg) species as compared to their predators. It seems logical that larger prey animals (or those with better defenses or escape ability) respond to a wider variety of chemical signals. Smaller, or more vulnerable, animals like murids should fear almost any sulfur-rich or nitrogen-containing odor regardless of the type of predator that produced it. There is a question whether a prey (e.g., a rat) should be more discriminating and should respond to single chemical compounds indicating a feline or canine, or whether a kangaroo should respond to canine, but certainly not, feline compounds. Larger prey, or more capable defenders, may also be more discriminating of additional compounds of the odor, such as the concentration ratio (intensity) of meat metabolites, an honest advertisement that a predator has previously consumed prey.

In situations where a prey survives the initial impact of Europe-originated predators, a predator may act as a strong selective agent for prey to develop strategies to manage predation risk. However, Australian studies on the use of unknown predator odor cues by mammalian prey species produced contradictory results. According to Woolhouse and Morgan (1995), some native species avoid the odors of all predators, but these native species were small and fall into the highest risk category for predation (Table 1, risk category 4). Data published by Nersesian et al. (2012) and Spencer et al. (2014) supported this finding. Other species appear to respond only to odors of native predators (Dickman, 1993). In some cases, native mammals show no evident avoidance of the odors of native and introduced predators (Blumstein et al., 2002).

Since the establishment of foxes to Australia in the 1870s, these predators have been linked to a local loss and regional extinction of several small to medium-sized mammals and some other vertebrates (Burbidge and McKenie, 1989). Two studies should be particularly emphasized. In 1998, Banks reported on the responses of wild Australian bush rats (*Rattus fuscipes*) to the odor (fresh feces tainted with the urine) of the European fox (*Vulpes vulpes*). Trapping success of rats was compared between clean traps and traps scented with fox odor in winter, spring, and summer. Trapping success was statistically analyzed and no difference between scented and unscented traps was found. Bush rats behaved naïvely toward the predator odor (Banks, 1998). In a similar second study, Banks et al. (2003) analyzed trapping success again using bush rats, with traps scented with dog feces and unscented traps. Bush rats showed no aversion to dog fecal odors and entered unscented and dog-scented traps equally. The researchers concluded that this lack of response may be because rats do not identify fox or dog scats as a cue to predation risk. However, another interpretation for these findings was that animals may have been startled (fled) into traps instead of away from them (a common occurrence that has been corroborated by video evidence), and thus trap-presence should not be a clear indicator for lack of vigilance. This is because animals are often recruited toward a scent in order to investigate additional scent-related information. In another field study (Anson and Dickman, 2013), behavior and in addition glucocorticoid hormone levels were analyzed on the marsupial ringtail possum (*Pseudocheirus peregrinus*). Animals were exposed to fox-suppressed areas and to areas where foxes were abundant. Ringtails showed no physiological or behavioral differences between the two areas. This lack of response to the fox odor may represent complete naivité or strong rapid selection to the invasive predator. Or, based on the relative risks posed by a predator relative to the size of prey, it could reflect the animal's category of risk. For instance, ringtail possums are capable defenders and fit within category 3 (lethal predator but uncommon predation), and would likely require more information such as a composite cue with direct predator presence in the immediate vicinity, or predator cues from a more threatening predator or group of counter-marking predators, before retreating from a preferred food patch. All these findings are somehow surprising, since fox feces is a very powerful odor to elicit escape reactions in European rodents. Mice (highest risk of lethality with predation common; category 4) would be more likely to be wary of any predator scent, regardless of the composite nature of timestamp of delivery. Macropods, the largest of the marsupials, would be the most selective in which predator scents to avoid. This may help explain why macropods can differentiate among risks posed by coyotes, domestic dogs, and dingo—with the primary avoidance response being to urine by dingoes.

Kovacs et al. (2012) reported on two common species of Australian small mammals (bush rat, *Rattus fuscipes*, and brown antechinus, *Antechinus stuartii*) that have persisted for over a century in the presence of the European fox). No difference in prey abundance in sites with high and low fox activity was found. However, survival of the bush rat was almost two-fold higher where fox activity was low. The conclusion of the authors was that populations of both species perform better where the activity of the predator is low. Interestingly, juvenile, but not adult rats, avoided fox odor on traps more strongly where fox activity was high than where it was low, but neither adult *R. fuscipes* nor *A. stuartii* responded differently to different levels of fox activity. Avoidance of fox odor declined over time.

In most areas of Australia, kangaroos enter farming areas and compete with farm animals for food, and are the primary selective agent in shaping forest rehabilitation following fire or anthropogenic disturbance, e.g., the kangaroo palate determines the composition of plant rehabilitation. As a consequence of this behavior, researchers have sought methods to influence food patch selection such that kangaroos would leave food patches with vulnerable, moist seedlings in exchange for mature forage that could compensate following herbivory. They tested the effectiveness of the urine of dingoes and non-native predators like coyotes (*Canis latrans*) to protect farming areas. When they experimentally deployed recent voids of dingo urine, kangaroos (*Macropus* spp.) were highly aroused and fled, some in excess of 50 m from the odor source (Parsons and Blumstein, 2010a). When they presented the novel coyote urine, kangaroos (large prey animals category 3) did not flee, but rather investigated the new smell—possibly to determine whether the scent conveyed enough risk to forgoing a feeding opportunity. This experiment was repeated in Tasmania (Parsons and Blumstein, 2010b) where wallabies (*Macropus rufogrisius*), pademelons (smaller macropods, *Thylagale stigmatica*) and brushtail possums (*Trichosuras vulepcula*) had never been exposed to dingoes (category 2). The outcomes, however, were similar in that all three small species (usually in the high risk categories) avoided the dingo scent (Parsons and Blumstein, 2010b). These outcomes may have been influenced because the dingoes had been regularly fed kangaroo carcasses prior to collection. While attempting to “synthesize” the urine to assist land rehabilitation, the authors determined that aged urine had completely lost its effectiveness (Figure 1) to such a degree that it could actually be used to attract the same animals the fresh urine had once repelled (Parsons et al., 2012). Again, it is assumed this “attraction” is based on attempts by the discriminating animal to obtain further information to warrant a behavioral response.

Kangaroos are both large in comparison to their predators and mobile, and should have a higher level of confidence that a specific chemical represents tangible risks before making a decision to forgo feeding. Therefore, kangaroos should be more discriminating than smaller mammals. This approach was supported when Cox et al. (2010), who learned that kangaroos were not repelled by carnivorous Tasmanian devils (*Sarcophilus harrisii*, a small predator); the authors reversed the outcomes: they fed kangaroo meat to Tasmanian devils, and the kangaroos fled.

Interestingly, Cox et al. (2014) were able to generate a similar response from an exotic non-native species, the tiger (*Pandera tigris*). When fed on kangaroo meat, tiger feces became an effective repellent for kangaroos, sometimes generating an “area-effect.” More recently, southern hairy-nosed wombats (*Lasiorhinus latifrons*) have been found to avoid digging in the area where dingo urine or feces had been deployed. Wombats remained in the area, as indicated by fresh tracks, but chose to dig tunnels in areas farther from dingo odors. Similar experiments with dingo urine were performed in Tasmania. Dingoes never entered Tasmania, but it was found that during the trials with natural dingo urine, supplemented with a gel, 78% of wallabies and 80% of possums were repelled (Macey, 2008). The authors again concluded that the smaller prey animals in higher risk categories may have been more sensitive and less discriminating to an unfamiliar predator. Unfortunately, the information about 200 chemical ingredients of dingo urine have not yet been published. Therefore, a comparison with the urine of old world carnivores is not available.

An examination of Table 1 suggests that the only category 2 interactions where a significant prey response was recorded, occurred from exotic—but large or pack-hunting predators—the tiger and the dingo. Whereas, 14 of the 18 occasions where category 3 or 4 interactions were inferred related in some level of aversion (Table 1). The remaining variation may be explained by a combination of single molecule deterrents being trialed such as the domestic dog synthetic used by Ramp et al. (2005), unique response variables such as measuring levels of novel food that has been placed in close proximity to the predator odor—where mixed plumes may conflate the identification of each molecule—or the level of preservation of frozen or partly degraded scents.

At least some prey species do not necessarily escape when a predator odor is encountered. This has been very convincingly shown in an open environment with the spinifex hopping-mouse (*Notomys alexis*), an Australian desert rodent. Spencer et al. (2014) tested the foraging and movement responses of the rodent to non-native predator (fox and cat) urine odor. Urines were collected from a fox just killed prior to urine sampling and stored at ~1°C until use; cat urine was obtained from euthanized cats. (Unfortunately no information is given in the paper for how long the urines were stored.) Rodents did not respond to these predator odors as one might have expected. Experience with an unknown predator and the stimuli emitted by the predator are for sure decisive for a balanced interaction between predators and prey (Anson and Dickman, 2013). Even in a fish predator, experience, and feeding history determines prey behavior and survival (Lönnstedt et al., 2012).

## Conclusions

Based on the lessons we have learned from the recent literature, particularly from field trials in Australia, we now have a heightened awareness of the following three factors that may influence reported outcomes:

1. Complexity of the molecular signal: Several studies have tried to find universal carnivore signals that, when received by a potential prey species, will be adequately responded to, even when the prey species never had encountered that predator or its odor before. Indeed, there are some volatile compounds found in many carnivores that elicit defensive and/or even fear reactions in a prey. Almost any sulfur-rich or nitrogen-containing compound elicits such behaviors regardless of the predator source. Generally, these single compounds are not effective alone (except TMT), but require other accompanying molecules to gain efficiency. Most often, single volatile substances convey very limited information about the predator. In contrast, arrays of different volatile compounds may convey more relevant information to the receiver, for example, the type of food the predator has consumed.
2. Chemical stability of the signal: A major consideration in all predator-prey studies is the stability of the chemical compounds and thus stability of the message. As demonstrated, aging of an odor bouquet could result in a modified information or even loss of the message.
3. Variable risk posed by the predator as compared to the prey: When evaluating the effectiveness of an odor used as a repellent, the size, and defensive abilities of the prey species has to be taken into account. In particular, Cox et al. (2010) showed that the largest macropods, Eastern gray kangaroos, will ignore recently-voided urine from the non-threatening, small Tasmanian devil. However, this wild-type response (e.g., ignoring small predators) was changed when the risk category was reversed by adding kangaroo meat to the predator diet. And this helped support our position that single molecule scents are less likely to be effective in deterring large herbivores, because larger animals may require more biologically relevant information than what a single molecule can provide. The Australian studies also demonstrate that not all potential prey species respond to unknown carnivore odors (not even to fox feces containing TMT) with defensive behaviors. Obviously they do not identify innately predator scents as a cue to predation risk; some even will show odor exploration behaviors when an unknown odor is presented. However, some species originally ignoring unknown predator odors learn to associate such odors over time, especially after the predator has preyed conspecifics.

### Conflict of interest statement

The authors declare that the research was conducted in the absence of any commercial or financial relationships that could be construed as a potential conflict of interest.
